# Clinically Relevant Anatomical Variations in the Brachial Plexus

**DOI:** 10.3390/diagnostics13050830

**Published:** 2023-02-22

**Authors:** Niki T. Patel, Heather F. Smith

**Affiliations:** 1 Arizona College of Osteopathic Medicine, Midwestern University, Glendale, AZ 85308, USA; 2 Department of Anatomy, College of Graduate Studies, Midwestern University, Glendale, AZ 85308, USA; 3 School of Human Evolution and Social Change, Arizona State University, Tempe, AZ 85287, USA

**Keywords:** anatomical variation, brachial plexus, neuralgia, thoracic outlet syndrome

## Abstract

Anatomical variation in the brachial plexus may result in a variety of clinically relevant patterns, including various neuralgias of the upper extremity and differing nerve territories. Some conditions can be debilitating in symptomatic patients, resulting in paresthesia, anesthesia, or weakness of the upper extremity. Others may simply result in cutaneous nerve territories that deviate from a traditional dermatome map. This study evaluated the frequency and anatomical presentations of a large number of clinically relevant brachial plexus nerve variations in a sample of human body donors. We identified a high frequency of various branching variants, of which clinicians, especially surgeons, should be aware. The medial pectoral nerves in 30% of the sample were found to originate from either the lateral cord, or both the medial and lateral cords of the brachial plexus rather than exclusively from the medial cord. The dual cord innervation pattern greatly increases the number of spinal cord levels traditionally believed to innervate the pectoralis minor muscle. The thoracodorsal nerve arose as a branch of the axillary nerve 17% of the time. The musculocutaneous nerve sent branches to the median nerve in 5% of specimens. The medial antebrachial cutaneous nerve shared a common trunk with the medial brachial cutaneous nerve in 5% of individuals and derived from the ulnar nerve in 3% of specimens.

## 1. Introduction

The brachial plexus is a crucial nerve network located in the upper extremity that is formed by the anterior rami of C5-T1 nerves. Recent studies have revealed that the brachial plexus is a far more variable anatomical structure than has traditionally been recognized [1,2,3,4,5,6,7,8,9,10,11,12,13,14,15,16,17,18,19,20,21,22,23,24,25,26,27,28,29]. The regular composition of trunks occurs in approximately 84% of studied cases. The divisions form cords in a classic pattern in 96% of cases. The terminal branches of the brachial plexus have been found to be more variable than the trunks and cords, retaining a classic arrangement in approximately 75% of cases [30].

Nerve branching variations may be associated with various upper extremity neuralgias and other clinical presentations [21,22,30,31,32]. Circumscapular pain has been investigated as resulting from entrapment of the dorsal scapular and long thoracic nerves, secondary to brachial plexus piercing variation [32]. Furthermore, research has been collected on general differences in brachial plexus roots, discovering that most of the variations occurred unilaterally and almost exclusively on the left side [21]. It was discovered that variations resulting in nerve branches coursing through the scalene musculature were quite common [21]. These piercing variants may be diagnosed via ultrasonography and are associated with higher risks of thoracic outlet syndrome symptoms [22].

Recently, a particular set of brachial plexus nerve variants was proposed to have clinically relevant results or symptoms [9]. Connections between nerves, for example, a proximal connection between the medial brachial cutaneous and medial antebrachial cutaneous nerve may result in overlapping sensory nerve territories. As clinicians often use classic dermatome maps for diagnostic purposes, such deviations from normal cutaneous innervation could result in misinterpretations in cases of nerve symptoms.

Similarly, the classic anatomical pattern presented in most medical school anatomy textbooks is that the medial pectoral nerve arises from the medial cord, while the lateral pectoral nerve arises from the lateral cord. However, recent studies have suggested that the pattern may be more complicated [26,33]. Some individuals possess an ansa pectoralis, in which the medial and lateral pectoral nerves connect and exchange fibers [33]. A dual cord origin suggests that the pectoral musculature may receive innervation from a far wider range of spinal cord levels (C5-T1) than is typically recognized [34]. This pattern has implications for the understanding and treatment of pectoral musculature deficits, as lesions to almost any part of the plexus would be expected to have at least minor effects on the strength of the pectoral girdle. However, there is conflicting information about how frequent these variations may be [26,33,34,35,36,37].

Similarly, if the ulnar nerve were to receive communicating branches from the lateral cord or the median nerve from the lateral cord, the myotomes associated with the nerves would differ from the classically understood patterns. In each case, lesions proximal to the peripheral nerve could have unexpected consequences that may lead to misdiagnoses. A similar situation could arise if the thoracodorsal nerve originated from the axillary nerve. A fracture to the surgical neck of the humerus commonly affects the axillary nerve, resulting in difficulty for the patient in abduction of the glenohumeral joint, and anesthesia or paresthesia in the skin of the lateral shoulder [36]. However, if the thoracodorsal nerve branched off the axillary nerve distal to such damage, the resulting symptoms would likely be expanded to include weakness of the latissimus dorsi and a reduced ability to adduct, medially rotate, and extend the arm [36]. Similarly, while a median nerve that received communicating branches from the musculoskeletal nerve might not have dramatic clinical symptoms in a patient without lesions, any damage to the latter could affect the nerve territory of the former. In all cases, nerve variants could cause unexpected presentations in cases of lesions, which may result in misdiagnoses.

Recent meta-analyses have also revealed substantial variation in reported frequencies of variations among studies, suggesting that regional differences may exist [35]. Only three of the seventy-five (4%) studies considered by Benes and colleagues [35] included cadavers from the United States. Previous studies were heavily skewed towards representation from Asia (75%), especially India. Thus, there is still a paucity of available data on these brachial plexus variants in North American populations.

With previous studies in mind, we will integrate the results with newly collected data to better understand the unique clinical implications of additional anatomical variations in the brachial plexus, placing heavy emphasis on thoracic outlet and associated syndromes. The focus of the current project was to thoroughly study these deviations from “normal” in the brachial plexus in a North American sample and investigate how they may be correlated with a plethora of clinical conditions. North American populations have been historically understudied regarding brachial plexus variations, as the vast majority of such studies have been conducted on populations in Asia and the Middle East. Thus, the percentage of nerve variations in North American populations is largely unknown. Of particular interest is the role that this complex network of nerves plays in traumatic injuries to the neck leading to anesthesia, paresthesia, and weakness in the upper extremities and hands.

The purpose of this study is to collect data from a sample of North American human cadavers to observe, document, and quantify the frequency of potentially clinically relevant anatomical variations in the branching patterns of the brachial plexus in a North American sample. The project tracked the routes of anomalies, paying particular attention to areas in which impingement/compression of the nerve is likely. As data and images of the brachial plexus nerve branches reaching their target tissues are collected, the variants were compared to the typical anatomical arrangement with the aim of determining if these alterations in nerve course could be relevant to neuralgias of unknown etiology in the upper extremity.

## 2. Materials and Methods

### 2.1. Samples

For this study, a total of 39 previously dissected cadaveric specimens from the Anatomy Department at Midwestern University were analyzed from February to July 2022. The sample consisted of twenty females and nineteen males. Analysis required meticulous cleaning of the brachial plexus, specifically its cords and terminal branches. Prior to data collection, the “M” of the brachial plexus, which is composed of the musculocutaneous, median, and ulnar nerves, was assessed to determine suitability of the body donor for study. Those donors with severely damaged brachial plexus nerves or pathological anomalies were excluded from the study. For included specimens, additional dissection and cleaning was conducted to improve the visual field for pertinent nerves and to enable tracing the nerves from their origin to target. The use of student-dissected cadavers limited the available intact anatomy to those variations listed in Table 1. All the specimens were coded by both researchers (NTP and HFS). This study was determined to be IRB-exempt by the Midwestern University Institutional Review Board (#AZ 1354).

### 2.2. Data Collection and Analysis

Each cadaver was scored separately on the left and right sides because the anatomy of the brachial plexus is known to vary bilaterally, e.g., [21]. Branching variants were investigated that have been hypothesized previously to have ramifying clinical symptoms [34] and were intact on the available sample of student-dissected cadavers. On each side of the specimen, the nerves that compose the “M”, plus the medial brachial cutaneous nerve, medial antebrachial cutaneous nerve, medial pectoral nerve, and thoracodorsal nerve were all followed from their origin to insertion points (Table 1). Variations in the typical “M” branching pattern were noted, paying particular attention to the meeting point of both medial and lateral cords to form the median nerve. In addition, both the medial brachial cutaneous and medial antebrachial cutaneous nerves were followed back to their origin, typically off the medial cord, and any modifications in this branching pattern were documented. Next, alterations in the origin of the medial pectoral nerve were investigated using the pectoralis minor muscle as a key landmark in identifying the correct nerve. Specimens in which the medial pectoral nerve did not originate from the medial cord directly were further inspected. Finally, the latissimus dorsi muscle was utilized to locate the thoracodorsal nerve, which was then traced along its route to determine whether it originated from the posterior cord as normal or whether a variation was present. True prevalence values were calculated for each variant by dividing the number of affected limbs by the number of limbs that could be scored for that variant. Upon collecting these data, a Chi-squared analysis was performed in SPSS 21 (IBM Corp.) to determine whether the distribution of the variables differed significantly between males and females or between left and right sides.

## 3. Results

### 3.1. “M” Branching

**Figure 1:**
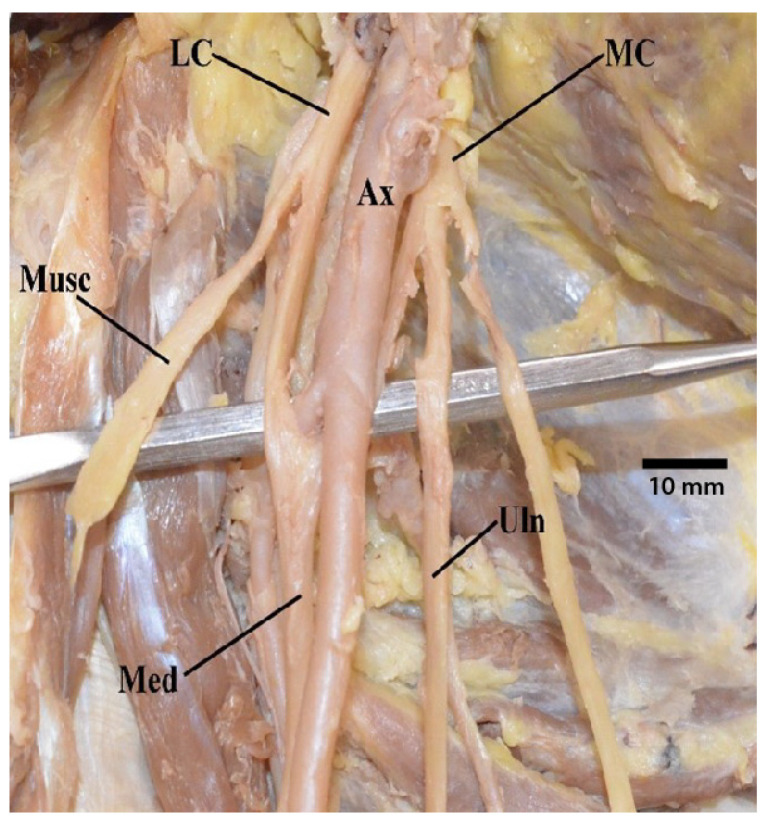
Photograph of the axillary artery located more anteriorly and positioned within the contents of the brachial plexus “M”. Abbreviations: Ax = axillary artery; LC = lateral cord; MC = medial cord; Med = median nerve; Musc = musculocutaneous nerve; Uln = ulnar nerve.

**Figure 2:**
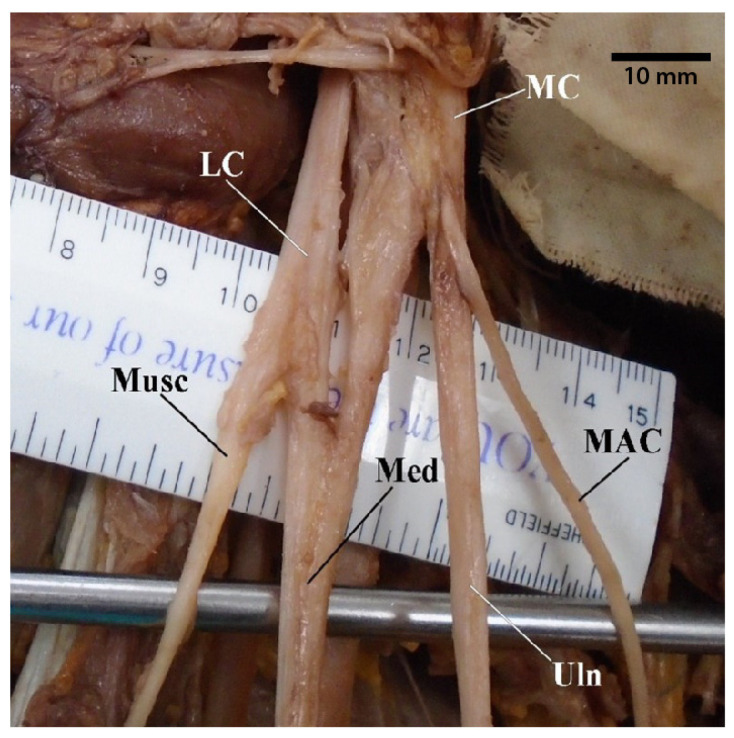
Photograph of a variant in which the brachial plexus “M” is asymmetrical but presents otherwise with its typical contributions and branches. Abbreviations: LC = lateral cord; MAC = medial antebrachial cutaneous nerve; MC = medial cord; Med = median nerve; Musc = musculocutaneous nerve; Uln = ulnar nerve.

Out of our cadaveric samples with intact brachial plexuses, 29 of the donors presented with classic “M” branching (Table 2 and Table S1). However, even among these normal contributions, in five specimens (6.6%), the axillary artery was positioned more anteriorly and intertwined with the contents of the “M”, referred to as a superficial brachial artery (Figure 1). Additionally, there were four specimens in which the “M” presented with its typical contributions but was asymmetrical due to abnormalities in how proximal or distal certain nerves were bifurcating (Figure 2).

**Figure 3:**
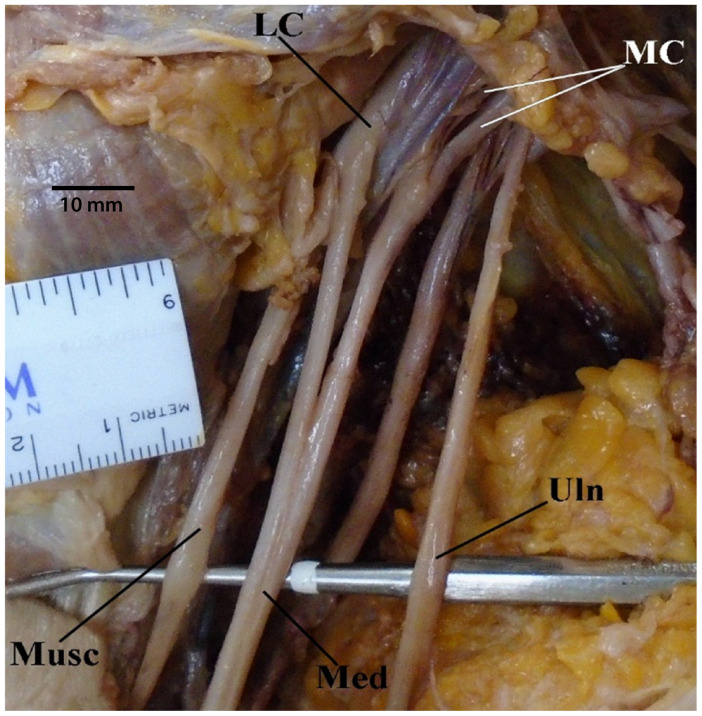
Photograph of the anatomical variant in which the median nerve is formed by two branches from medial cord and one branch from lateral cord. Abbreviations: LC = lateral cord; MC = medial cord; Med = median nerve; Musc = musculocutaneous nerve; Uln = ulnar nerve.

The remaining individuals were found to have abnormal “M” branching. Most commonly, the median nerve was formed via two branches from the lateral cord and one branch from the medial cord; this was discovered to be the case directly in 14 cases and indirectly in 15 cases, in which the lateral cord contributed a branch that dove into the medial cord’s contribution to the median nerve. In 29 total specimens, the median nerves were composed of two branches from the lateral cord and one branch from the medial cord (see Section 3.7). Contrastingly, the median nerve was formed by two branches from the medial cord and one branch from the lateral cord only once (Figure 3).

**Figure 4:**
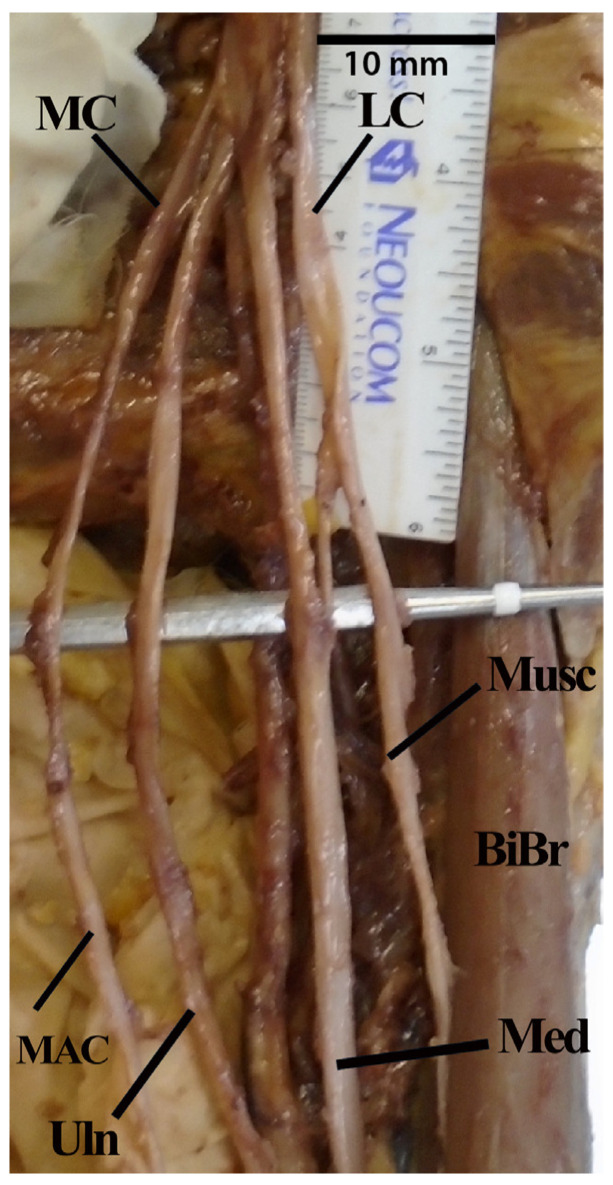
Photograph of musculocutaneous nerve piercing biceps brachii rather than coracobrachialis. Abbreviations: BiBr = biceps brachii muscle; LC = lateral cord; MAC = medial antebrachial cutaneous nerve MC = medial cord; Med = median nerve; Musc = musculocutaneous nerve; Uln = ulnar nerve.

**Figure 5:**
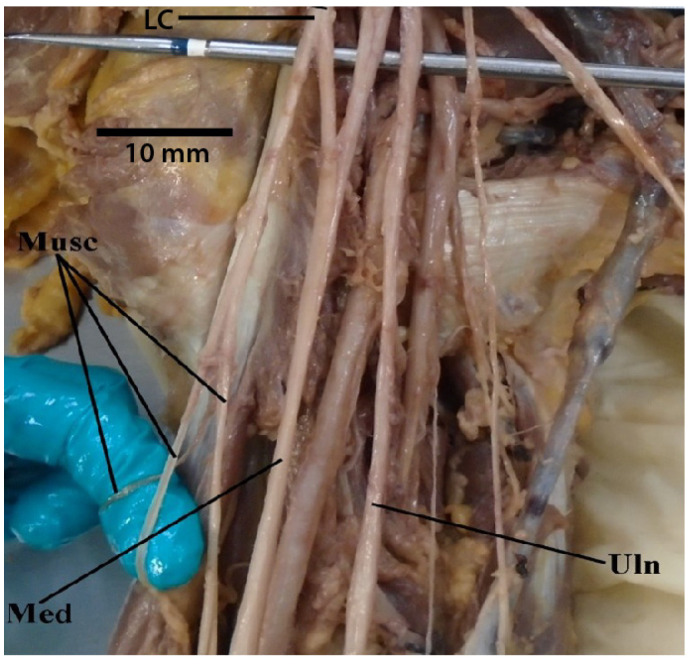
Photograph of multiple proximal branches of the musculocutaneous nerve. Abbreviations: LC = lateral cord; Med = median nerve; Musc = musculocutaneous nerve; Uln = ulnar nerve.

Two other frequent atypical branching patterns involved the musculocutaneous nerve. Nine examples were found of the musculocutaneous nerve not piercing through coracobrachialis and the nerve route was much longer than normal; instead, the nerve pierced through brachialis and/or biceps brachii muscle (Figure 4). The other disparity pertaining to the musculocutaneous nerve was multiple proximal branches of this nerve emanating prior to the nerve diving into the anterior arm muscles, which was also noted on nine separate occasions (Figure 5).

The remaining three branching patterns were far less common and only observed one time each—a medial antebrachial cutaneous nerve arising from the medial cord’s contribution to the median nerve, a crisscrossing connection between the medial and lateral cords proximal to the “M”, and the fusion of medial and lateral cords proximal to the “M”.

### 3.2. Common Trunk of Medial Brachial Cutaneous Nerve and Medial Antebrachial Cutaenous Nerve

**Figure 6:**
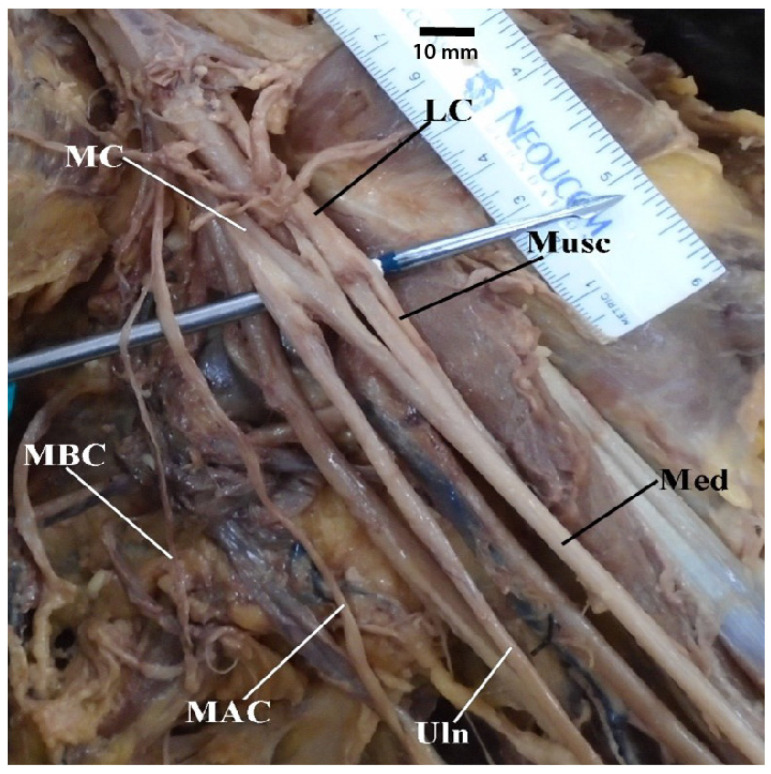
Photograph showing a common trunk of medial brachial cutaneous nerve and medial antebrachial cutaneous nerve. Abbreviations: LC = lateral cord; MAC = medial antebrachial cutaneous nerve; MBC = medial brachial cutaneous nerve; MC = medial cord; Med = median nerve; Musc = musculocutaneous nerve; Uln = ulnar nerve.

There were four occurrences in which the medial brachial cutaneous and medial antebrachial cutaneous nerves arose from a common trunk coming off the medial cord (Figure 6).

### 3.3. Medial Antebrachial Cutaneous Nerve Arising from Inferior Trunk or Ulnar Nerve

**Figure 7:**
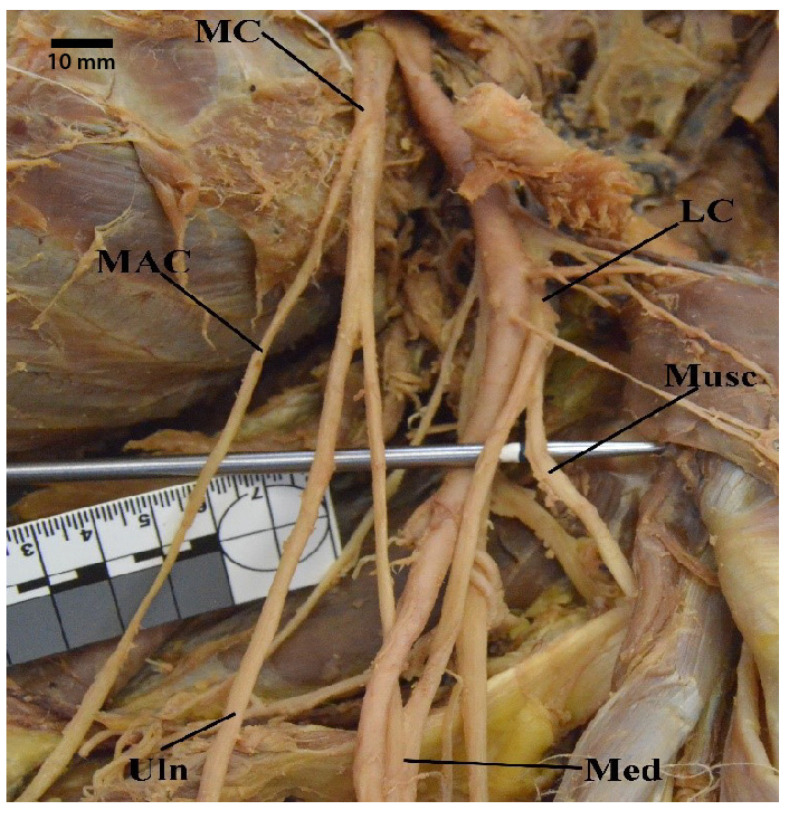
Photograph of an individual in which a long medial cord gives rise very proximally to medial antebrachial cutaneous nerve. Abbreviations: LC = lateral cord; MAC = medial antebrachial cutaneous nerve; MC = medial cord; Med = median nerve; Musc = musculocutaneous nerve; Uln = ulnar nerve.

**Figure 8:**
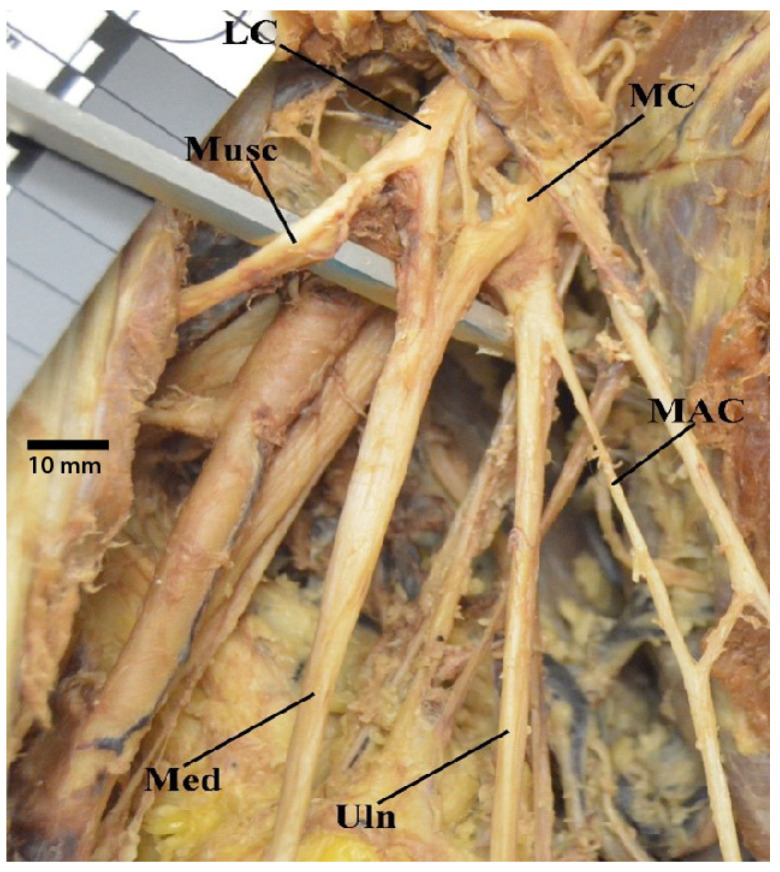
Photograph demonstrating a medial antebrachial cutaneous nerve arising from the ulnar nerve. Abbreviations: LC = lateral cord; MAC = medial antebrachial cutaneous nerve; MC = medial cord; Med = median nerve; Musc = musculocutaneous nerve; Uln = ulnar nerve.

In this sample, there were no cadavers in which the inferior trunk gave rise to the median antebrachial cutaneous nerve. However, there was one instance in which a long medial cord gave rise very proximally to the medial antebrachial cutaneous nerve (Figure 7). There were two cases in which the medial antebrachial cutaneous nerve branched directly off the ulnar nerve instead of the medial cord (Figure 8).

### 3.4. Medial Pectoral Nerve Receives Contributions from Both Medial and Lateral Cords or Just Lateral Cord

**Figure 9:**
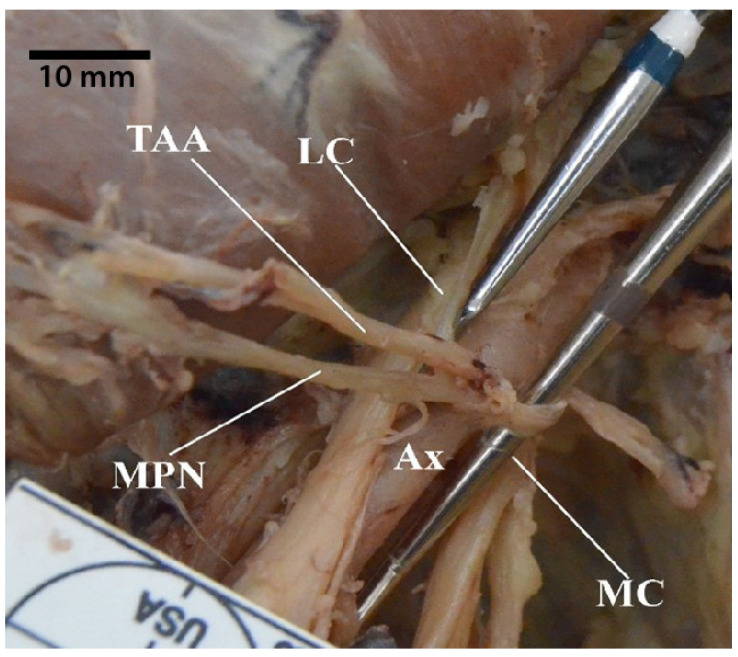
Photograph showing the medial pectoral nerve receiving contributions from both medial and lateral cords with the pectoral branch of thoracoacromial artery travelling in between the two contributions. Abbreviations: Ax = axillary artery; LC = lateral cord; MC = medial cord; MPN = medial pectoral nerve; TAA = thoracoacromial artery (pectoral branch).

**Figure 10:**
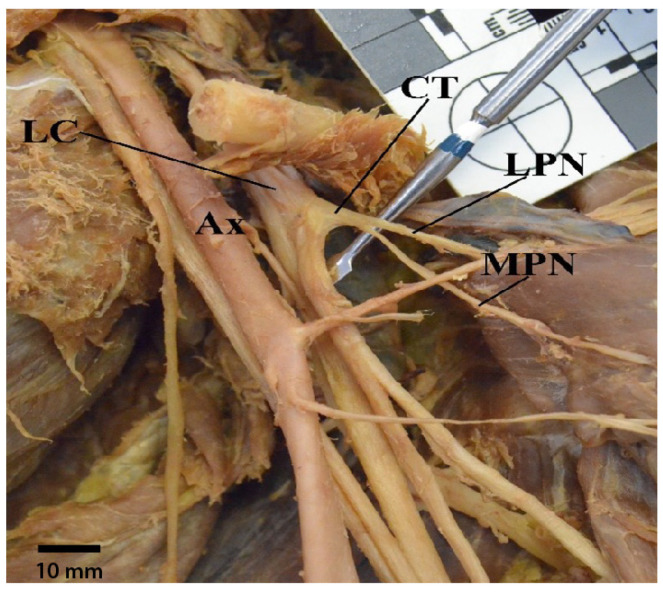
Photograph showing an example of a medial pectoral nerve sharing a common trunk with lateral pectoral nerve. Abbreviations: Ax = axillary artery; CT = common trunk; LC = lateral cord; LPN = lateral pectoral nerve; MPN = medial pectoral nerve.

There were seven occurrences in which the medial pectoral nerve received contributions from both medial and lateral cords. Of these seven, in one cadaver, the pectoral branch of the thoracoacromial artery traveled between the two contributions from each cord (Figure 9). Further, there were six additional cases in which the medial pectoral nerve came from the lateral cord via a common trunk with the lateral pectoral nerve (Figure 10). Lastly, one of our specimens presented with a split musculocutaneous nerve and its medial pectoral nerve appeared to come off the second branch of the musculocutaneous nerve.

### 3.5. Thoracodorsal Nerve Originating from the Axillary Nerve

**Figure 11:**
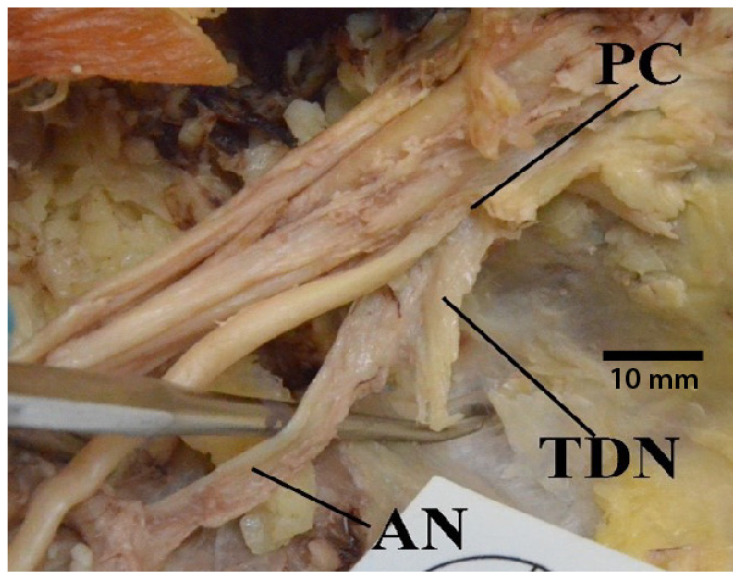
Photograph of the variant in which the thoracodorsal nerve originates from the axillary nerve. Abbreviations: AN = axillary nerve; PC = posterior cord; TDN = thoracodorsal nerve.

There were eight different occasions on which the thoracodorsal nerve originated from the axillary nerve, instead of from the posterior cord (Figure 11). Additionally, there were several donors in which the thoracodorsal nerve branched off the posterior cord; however, the location was exactly at the point where the posterior cord bifurcated into the radial and axillary nerves, making these specimens ambiguous with regards to the variable studied.

### 3.6. Ulnar Nerve Receiving Communicating Branches from the Lateral Cord

**Figure 12:**
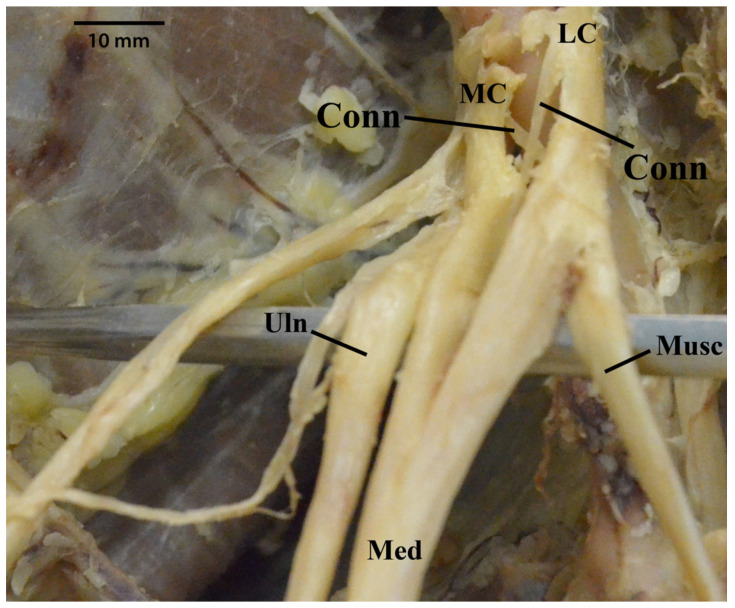
Photograph demonstrating a crisscrossing connection between the medial and lateral cords proximal to the “M”. Abbreviations: Conn = connections between lateral cord and medial cord; LC = lateral cord; MC = medial cord; Med = median nerve; Musc = musculocutaneous nerve; Uln = ulnar nerve.

Unfortunately, this variable was one of the most difficult to distinguish accurately. On five separate occasions, it was definitively concluded that the ulnar nerve received communicating branches from the lateral cord. For example, one cadaver emerged with a crisscrossing connection between the medial and lateral cords proximal to the “M”; therefore, we deduced that the ulnar nerve did, indeed, receive branches from the lateral cord via its overlapping connection (Figure 12). In another case, it was uncertain of whether the ulnar nerve was receiving branches from the lateral cord because there was an extra branch connecting the medial and lateral cords, which appeared to share a small connection with the ulnar nerve. In another case, one of the lateral cord’s contributions to the median nerve was also suspected of sending some fibers to the ulnar nerve.

### 3.7. Median Nerve Receiving Branches from the Musculocutaneous Nerve

**Figure 13:**
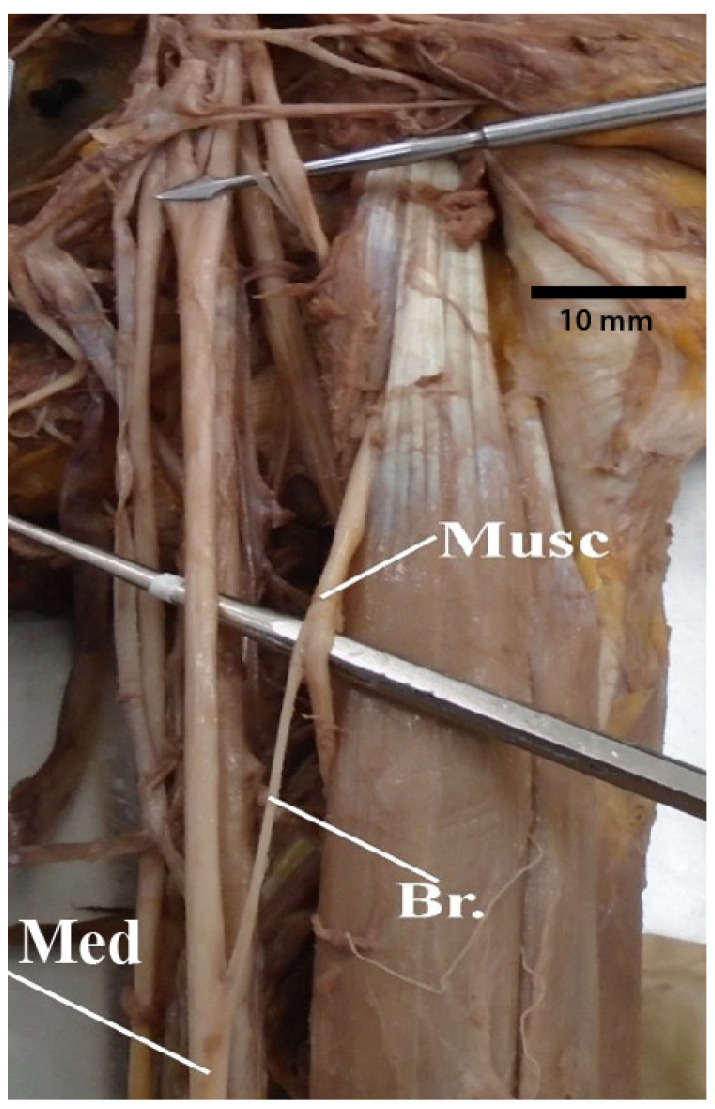
Photograph of the median nerve receiving branches from the musculocutaneous nerve. Abbreviations: Br. = branch from musculocutaneous nerve to median nerve; Med = median nerve; Musc = musculocutaneous nerve.

There were four different instances in which the median nerve received branches from the musculocutaneous nerve (Figure 13). Of these, half were due to the initial free portion of the musculocutaneous nerve being substantially longer than normal and not piercing through the coracobrachialis muscle. Instead, the nerve dived into the brachial musculature more distally than normal, piercing either the brachialis and/or biceps brachii muscle. This arrangement permitted closer access between the musculocutaneous and median nerves and resulted in a connection between the two nerves.

### 3.8. Median Nerve Receiving Branches from the Posterior Cord

While this variable was rare, it did occur in one individual, solely due to the fusion of the medial and posterior cords. This resulted in median nerve being composed of fibers from all three cords—medial, lateral, and posterior. Thus, in this specimen, the median nerve did indirectly receive branches from the posterior cord.

### 3.9. Results of Statistical Analyses

The chi-squared tests revealed no significant differences in the distribution of the tested brachial plexus branching variants. Similarly, no significant differences were observed in the variables between the left and right sides of the body.

## 4. Discussion

Anatomical variation is extremely relevant for any surgical or other invasive medical procedures that rely upon an assumption of classical human anatomy. It is crucial that clinicians, especially surgeons, be aware of how common clinically relevant nerve variations may be in order to avoid compromising patient care and to improve patient outcomes.

One of the most common nerve variations revealed here was a medial pectoral nerve (MPN) that received fibers from both the medial and lateral cords of the brachial plexus (17%). This pattern may involve an ansa pectoralis, in which the medial and lateral pectoral nerves connect. Loukas and colleagues [33] reported a 28% occurrence of the ansa pectoralis in their sample. The ansa pectoralis arising has been described as originating from the deep branch of the middle pectoral nerve [33,34]. Terminal branches from the ansa pectoralis may innervate the pectoral muscles [36]. Another study reported that the medial pectoral nerve may connect to the anterior division of the upper trunk (5%), middle trunk (25%), or to a deep branch of the lateral pectoral nerve [37]. Interestingly, Benes et al. [6] categorized the medial pectoral nerve into five types based on origin: from a medial cord (90.9%), from C8 (<0.1%), from T1 (<0.1%), from the anterior division of a middle trunk (<0.1%), and from the anterior division of an inferior trunk (7.2%). They did not recognize a classification involving contributions to the medial pectoral nerve from the lateral cord, although the anterior division of the middle trunk converges to form the lateral cord. Their results contrast with that of Porzionato et al. [26], who found that the medial pectoral nerve only displays a classic branching pattern from the medial cord in 49.3% of cases. Benes et al. [6] ascribe these differing frequencies to methodological differences between the studies.

The thoracodorsal nerve originated from the axillary nerve in 17% of specimens. This frequency is far higher than the 4% reported by Benes et al. [6]. However, within the included studies of the Benes meta-analysis, two individual studies reported comparable percentages to ours. In Al-Hubaity et al. [38], 10/50 cadavers (20%) had demonstrated a thoracodorsal nerve branching off the axillary nerve; and in Rastogi et al. [39], it was 17/74 (23%). Therefore, it appears that this variation may vary more widely across samples compared to other included variants.

We identified a common trunk of the medial brachial and antebrachial cutaneous nerves in 5% of cases, similar to the 3% identified by Benes et al. [6]. Alternatively, we found that the medial antebrachial cutaneous nerve arose from the ulnar nerve in 3% of specimens. The latter was not a condition recognized by Benes et al. [6]; thus, its previously documented frequency is unknown.

The median nerve had a high percentage of variations, consistent with previous studies. In particular, it received multiple contributions from the lateral cord in 43% of cases. However, a confounding factor with this variable is that during student dissections, occasionally some of the epineurium surrounding the cords may be inadvertently removed. While we attempted to identify any such overly dissected specimens, it is conceivable that some of the seemingly duplicated lateral cord contributions were initially single contributions that had been artificially separated during the dissection process. We also observed the musculocutaneous nerve to supply branches to the median nerve in 5% of specimens. This variable may be comparable to the variable of multiple contributions from the lateral cord documented by Benes et al. [6]. While we defined the musculocutaneous nerve as originating from the point at which the lateral cord sends its main contribution to the median nerve, an alternate definition is that the mucocutaneous nerve begins only after all the lateral cord’s contributions have been sent to the median nerve. Under the latter definition, the 8% of multiple lateral cord contributions of Benes et al. [6] may be equivalent to our 5% musculocutaneous contributions. In 1% of specimens, the posterior cord contributed fibers to the median nerve. This scenario was not discussed in Benes et al. [6], although they did mention six unspecified cases of “other” variations. The musculocutaneous nerve was observed emanating multiple proximal branches in 11.8% of our sample. Surprisingly, these values are far lower than the 90% reported by previous studies [12,20,24]. However, the more interesting functional detail is not whether multiple branches of the nerve emerged, but their destination. This topic could be further explored in future studies.

We observed several cases (6.6%) in which the brachial artery coursed anterior to the median nerve, often referred to as a superficial brachial artery. This artery has been previously documented in 3.6–9.6% of the population [13,18,25]. While not a nerve variation per se, the relationship between the axillary artery and peripheral nerves of the brachial plexus can affect how the latter functions. If a superficial brachial artery compresses the median nerve, it could cause radiating pain, paresthesia, or weakness in the upper extremity.

The present study revealed that a sizeable percentage of the population may have nerve branching variants in the brachial plexus. Such nerve variants have particular clinical implications for thoracic outlet syndrome, a relatively common condition affecting up to 8% of the U.S. population [40]. In particular, neurogenic thoracic outlet syndrome results from the compression of brachial plexus nerves as they course into the upper extremity, resulting in pain, paresthesia, anesthesia, and even paresis [40,41,42]. Current diagnostic modalities are typically based on an underlying assumption of a classical anatomical relationship among the nerves of the brachial plexus; however, this is often not the case. Awareness of these nerve variations may improve patient outcomes. Nerve anomalies may also be visualized using ultrasound if a physician is aware to look for them in advance of a procedure.

This study contributes to the growing body of knowledge on anatomical variation in brachial plexus branching patterns. Previous studies have revealed “piercing variants” in which the roots or trunks of the plexus course through the bellies of the scalene musculature, which may predispose patients to TOS symptoms [21,22,32]. The current study expands upon these findings to reveal a high percentage of additional branching variations, which also necessarily alter the function of peripheral nerves in the upper extremity.

## 5. Conclusions

Several anatomical variations in the brachial plexus branching patterns were evaluated for the first time in a North American population. Only 40% of the sample presented with a classic “M” arrangement. The medial antebrachial cutaneous nerve shared a common trunk with the medial brachial cutaneous nerve in 5% of cases and originated from the ulnar nerve in 3% of specimens. An ansa pectoralis connecting the medial and lateral pectoral nerves was present in 16% of individuals. The thoracodorsal nerve arose from the axillary nerve in 17% of cases, the ulnar nerve received branches from the lateral cord in 8% of cases, and the musculocutaneous nerve gave fibers to the median nerve in 5% of cases. These findings demonstrate the relative high rates of variation in the branching patterns of the brachial plexus in a North American population and suggest that care should be taken in any medical or surgical procedures in this area, which may be based upon an assumption of normal anatomy.

## Figures and Tables

**Table 1:** Variables on clinically relevant brachial plexus branching variations collected in this study.

Variable
1. Is the “M” of the brachial plexus a classic anatomical arrangement?
2. Do the medial brachial cutaneous nerve and medial antebrachial cutaneous nerve arise from a common trunk?
3. Does the medial antebrachial cutaneous nerve arise from the inferior trunk or ulnar nerve?
4. Does the medial pectoral nerve receive fibers from only the lateral cord or from both medial and lateral cords?
5. Does the thoracodorsal nerve originate from the axillary nerve?
6. Does the ulnar nerve receive communicating branches from the lateral cord?
7. Does the median nerve receive branches from the musculocutaneous nerve?
8. Does the median nerve receive branches from the posterior cord?

**Table 2:** True prevalence of brachial plexus branching variants identified in the present study.

Variant	Yes— Male	Yes— Female	Yes— Total
Classic M pattern	11/38 (29%)	18/38 (47%)	29/76 (38%)
Common trunk of MBC + MAC	1/38 (3%)	3/38 (8%)	4/76 (5%)
MAC from IT or ulnar n.	2/36 (6%)	0/33 (0%)	2/69 (3%)
MPN from MC + LC	4/23 (18%)	3/21 (14%)	7/44 (15.9%)
MPN from LC	1/23 (4%)	5/21 (24%)	6/44 (14%)
Thoracodorsal n. from axillary n.	6/25 (24%)	2/22 (9%)	8/47 (17%)
Ulnar n. rec bb. from LC	4/38 (11%)	2/38 (5%)	6/76 (8%)
Musculocutaneous n. bb to median n.	0/38 (0%)	4/37 (11%)	4/75 (5%)
PC gives bb. to median n.	0/38 (0%)	1/38 (3%)	1/76 (1%)

Abbreviations: bb. = branches; IT = inferior trunk; LC = lateral cord; MAC = medial antebrachial cutaneous nerve; MBC = medial brachial cutaneous nerve; MC = medial cord; MPN = medial pectoral nerve; n. = nerve; PC = posterior cord.

## Data Availability

All relevant data are provided within the paper or uploaded as Supplementary Table S1.

## Supplementary Materials

The following supporting information can be downloaded at: https://www.mdpi.com/article/10.3390/diagnostics13050830/s1, The following are available as supplementary files online—Table S1: Raw data file.
